# Microbial induced solidification and stabilization of municipal solid waste incineration fly ash with high alkalinity and heavy metal toxicity

**DOI:** 10.1371/journal.pone.0223900

**Published:** 2019-10-17

**Authors:** Ping Chen, Hao Zheng, Hui Xu, Yan-xu Gao, Xiao-qing Ding, Mei-ling Ma

**Affiliations:** School of Civil Engineering and Architecture, Zhejiang Sci-Tech University, Hangzhou, China; Gifu University, JAPAN

## Abstract

This paper presents an experimental study on the applicability of microbial induced carbonate precipitation (MICP) to treat municipal solid waste incineration (MSWI) fly ash with high alkalinity and heavy metal toxicity. The experiments were carried out on fly ashes A and B produced from incineration processes of mechanical grate furnace and circulating fluidized bed, respectively. The results showed that both types of fly ashes contained high CaO content, which could supply sufficient endogenous Ca for MICP treatment. Moreover, *S*. *pasteurii* can survive from high alkalinity and heavy metal toxicity of fly ash solution. Further, the unconfined compressive strength (UCS) of MICP treated fly ashes A and B reached 0.385MPa and 0.709 MPa, respectively. The MICP treatment also resulted in a reduction in the leaching toxicity of heavy metals, especially for Cu, Pb and Hg. MICP had a higher solidification and stabilization effect on fly ash B, which has finer particle size and higher Ca content. These findings shone a light on the possibility of using MICP technique as a suitable and efficient tool to treat the MSWI fly ash.

## Introduction

In recent years, incineration method has been widely applied to manage the municipal solid waste (MSW) in the world. Even though incineration is efficient for treating MSW, its major shortcoming is the large production of fly ash [1]. In China, 73.78 million tons of MSW was incinerated in 2016 (data from China Statistic Almanac), concomitantly producing 1.47~3.69 million tons of fly ash (2~5% of the MSW by weight [2]. Municipal solid waste incineration (MSWI) fly ash is classified as hazardous waste due to that the pollutants (e.g., heavy metals) exceed the limits of the identification standard for hazardous wastes. According to the existing legislation in most countries, proper treatment must be taken for the MSWI fly ash before its disposal in sanitary landfills [3].

MSWI fly ash can be disposed in several ways, including chemical stabilization, cement solidification and thermal separation [4]. The process of cement solidification is widely understood, easily available, and often attains an acceptable solidification of the materials and a good degree of fixation of heavy metals [5]. However, the volume of the solidified products usually increases considerably, leading to an increase in the cost of disposal [6]. The process of chemical stabilization results in little volume increase and a good stabilization of heavy metals [1, 7]. However, it is difficult to find a chelating agent completely efficient for a variety of heavy metals contained in MSWI fly ash [4]. Moreover, the cost is still too high for practical implementation [8]. The process of thermal separation of heavy metals by evaporation or vitrification at a high temperature has obvious shortcomings in the aspect of energy consumption and investment in equipment [9]. Therefore, it is necessary to seek technically efficient, cost-effective and environment-friendly methods for the treatment of MSWI fly ash.

Microbial induced calcite precipitation (MICP) seems to provide an alternative to solve this problem. The MICP process involves two main stages [10–11]. The first stage is the hydrolysis of urea [CO(NH_2_)_2_]. It is a process where urea is decomposed into NH_4_^+^ and CO_3_^2−^ under the catalytic action of urease produced from bacteria, as presented in Eq (1). The second stage is the precipitation of calcium carbonate crystals. This process happens as a consequence of the reaction between CO_3_^2−^ liberated from urea hydrolysis and Ca^2+^ released from a calcium source, as presented in Eq (2). In this process, the bacteria act as nucleation sites for the growth of calcite particles. As metal ions are bound to the bacterial cell wall as a result of the negative charge of the latter, this can result in the formation of crystals on the surface of the bacterial cell [12]. It is known that the bacterial properties (i.e., population, activity) can significantly influence the precipitation processes described above, and therefore, a suitable environment (i.e., temperature, pH, moisture) provided for bacteria is vitally important [13]. The induced calcite crystals are known to act as microbiological binder of cement based materials, which can form cohesive “bridges” between solid particles [14]. This process is similar to the mechanism of cement solidification. Along with the precipitation of calcium carbonate, heavy metal ions with radius close to Ca^2+^, such as Sr^2+^, Pb^2+^, Cd^2+^ and Cu^2+^, may be incorporated into the calcium carbonate crystal by substituting the Ca^2+^ in the lattice, or entering the interstice or defect of the crystal [15]. This process can strongly immobilize the heavy metals, which is similar to chemical stabilization. Therefore, MICP has emerged as a sustainable, eco-friendly approach for the solidification and stabilization of porous materials.

$$CO(NH_{2}{)}_{2}+2H_{2}O\overset{\mathrm{Urease}}{\to }CO_{3}^{2‐}+2NH_{4}^{+}$$ (1)

$$Ca^{2+}+CO_{3}^{2‐}\to \mathrm{CaC}O_{3}$$ (2)

In recent years, the MICP technology has been developed rapidly and been widely used in geotechnical and environmental engineering. A series of applications are situated in the field of bio-remediation. The bio-remediation includes the removal of heavy metals from contaminated soils [16–17], the control of internal erosion in gravel-sand/sand-clay mixtures [18–19], and the treatment of groundwater contaminated by heavy metals or radio nucleotides [20–22]. It should be noted that most of the previous researches mentioned above are carried out on the porous materials in conditions of pH 5–9 with certain limited types of heavy metal. However, as for MSWI fly ash, the pH value is in the range of 10–13 at liquid-solid ratio of 2.5–100 [8, 23]. Meanwhile, it contains multiple and uncertain types of heavy metals, the contents of some typical heavy metal ions such as Zn^2+^, Cu^2+^, Pb^2+^ are even over 500 mg/kg [3–4, 8, 23]. It is notable that MSWI fly ash creates a much more complicated biochemical environment for MICP. Therefore, it is scientifically valuable to assess the applicability of MICP to stabilize and solidify the MSWI fly ash with high alkalinity and heavy metal toxicity. However, few studies have been carried out to focus on this topic.

The primary objective of this paper was to explore the potential application of MICP in treating two types of MSWI fly ash from different incineration processes. Subject involves: a) physical and chemical properties of two types of fly ashes were analyzed to assess their substance basis and the necessity for MICP treatment; b) microbial concentrations in the bacteria-ash-water mixture were measured to assess the activity and fertility of microbe; c) MICP solidification and stabilization treatments were carried out, while leaching concentrations of heavy metals were tested to evaluate the stabilization degree of the fly ashes and unconfined compressive strength (UCS) and particle size distribution were tested to evaluate the solidification degree of the fly ashes.

## Material and methods

### Characteristics of MSWI fly ashes

Two types of MSWI fly ashes were sampled in this study, here named fly ash “A” and “B”. Fly ash A was collected from the MSWI plant with burning equipment of mechanical grate furnace, which is operated by Green Energy Environmental Protection Power Co., Ltd in Hangzhou, China. Fly ash B was collected from the MSWI plant with burning equipment of circulating fluidized bed, which is operated by Jinjiang Green Energy Co., Ltd in Hangzhou, China.

The following tests were carried out to characterize the two types of fly ashes. The particle size distribution was analyzed by the mechanical sieve method (diameter ≥ 0.075mm) and hydrometer method (diameter < 0.075 mm). The pH value was measured in the ash-water mixture with solid-liquid ratio of 1:10 (1 g:10 mL) by a pH meter. The chemical compositions were determined by an energy dispersive X-ray spectrometer (EDX, JSM-5610LV, Japan). The toxicity characteristic leaching procedure (TCLP) test, following China EPA method HJ/T 299–2007, was employed to evaluate the leaching properties of trace metals. The determined metal includes Cr, Ni, Cu, Zn, Cd, Hg, Pb. The metal was dissolved out using an extraction buffer of sulfuric acid—nitric acid solution with a pH of 3.2±0.05. In the leaching procedure, 150 g fly ash samples were mixed with the extraction solution in a solid-liquid ratio of 1:10. After that, the mixture was vibrated at a rotation rate of 30 rpm and a temperature of 23±2°C for 18 hours. The resulting solution was filtered and analyzed for metal content by using an inductively coupled plasma-mass spectrometer (ICP-MS, Agilent 7700X, China). The total metal content embodied in the fly ash was determined according to the China EPA method HJ/T 166–2004. The fly ash sample was firstly dissolved using the four acids method, and then analyzed with ICP-MS.

### Measurement of microbial concentration

The strain adopted in this study is *S*.*pasteurii*, which was obtained from the China General Microbiological Culture Collection Center (CGMCC). *S*.*pasteurii*, a high-performance urease-producing strain, is one of the most commonly used bacterium for carbonate precipitation [24–25]. The liquid medium for the cultivation of *S*.*pasteurii* contained 5 g/L of peptone, 3 g/L of beef extract and 20 g/L of urea.

The process for testing the concentration of *S*.*pasteurii* is presented as follows. The selected bacterial colony was added into the liquid medium with a volumetric ratio of 1:1000 to form an initial bacterial suspension. After that, the bacteria-ash- water mixture was prepared by mixing 15 g fly ash sample with 150 mL initial bacterial suspension. Subsequently, the mixture was placed statically for one hour, and 4 mL of the supernatant liquid was then extracted and filtered at 3 um to analyze the microbial concentration. After that, the mixture was placed in an incubator (MaxQ 4000, Thermo Fisher Scientific, USA) at shaking rate of 220 rpm and temperature of 30°C. After cultivating for 24 hours, the mixture was then placed for one hour and the supernatant liquid was sampled. Such repeated, five samples were finally obtained, which respectively stood for the culturing times of 0, 24, 48, 72 and 96 hours. The microbial concentration of samples was analyzed by measuring the OD600 value (optical density at 600 nm) using an ultraviolet-visible spectrophotometer (Model U-2800, Hitachi, Japan). The measured OD600 value of the sample without cultivation was taken as the base value. A reference test was also carried out, in which the fly ash sample was replaced by an identical amount of deionized water. Three parallel tests were carried out for each case, and the average values were used for the discussion of test results.

### Processes for solidification and stabilization treatments

Four cases of solidification and stabilization treatments were designed for fly ash A and B, as shown in Table 1. All cases were conducted on the ash-liquid mixture with a same solid-liquid ratio of 1: 0.3 but mixed with different types of liquids. Case 1 was set as a reference test, and the fly ash was mixed with deionized water, to study the self-cementation capacity of MSWI fly ash. In Case 2, the fly ash was mixed with deionized water containing 0.33 mol/L urea (i.e., urea solution), which was performed to study the effect of urea concentration on the self-cementation. Case 3 was mixed with bacterial suspension which contained 0.33 mol/L urea in the liquid medium, to characterize the bio-cementation capacity of MSWI fly ash after MICP treatment. The bacterial suspension was cultured in an incubator at shaking rate of 220 rpm and temperature of 30°C for 48 hours, which had an OD600 value of 0.174±0.032. Case 4 was conducted under the condition similar to Case 3, except for a higher urea concentration of 0.67 mol/L. It was performed to investigate the effect of urea concentration on the bio-cementation.

**Table 1:** Cases of solidification and stabilization treatments.

Case	Solid-liquid ratio (kg : L)	Liquid mixed
**Case 1: A1&B1**	1.0 : 0.3	deionized water
**Case 2: A2&B2**	1.0 : 0.3	0.33 mol/L urea solution
**Case 3: A3&B3**	1.0 : 0.3	bacterial suspension contained 0.33 mol/L urea
**Case 4: A4&B4**	1.0 : 0.3	bacterial suspension contained 0.67 mol/L urea

Note: A1, A2, A3, A4 refer to fly ash A, and B1, B2, B3, B4 refer to fly ash B.

**Fig 1:**
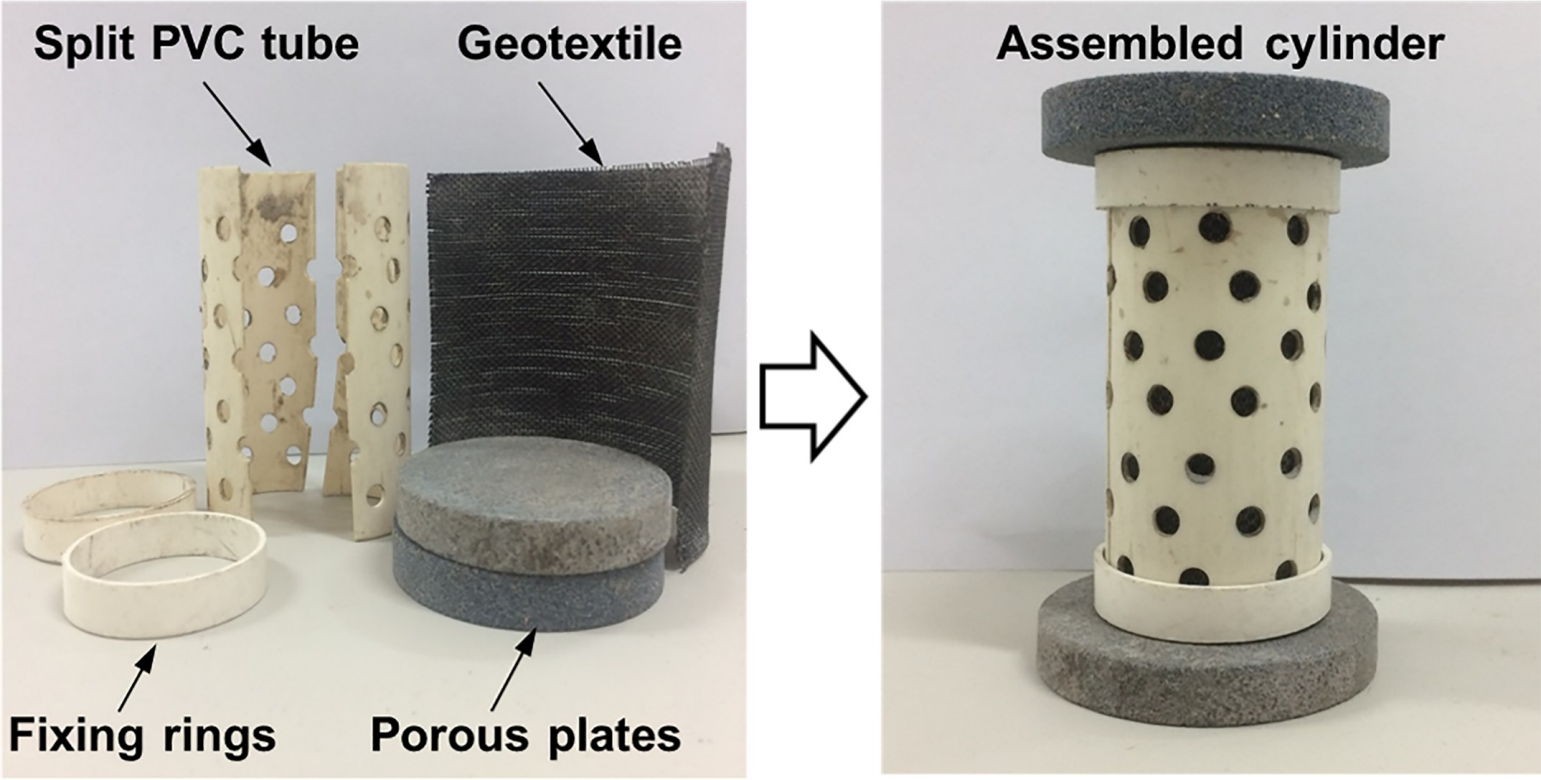
Cylindrical mold for MICP treated specimens.

Taking Case 4 for an example, the testing processes were presented as follows:

(1) The MSWI fly ash sample was dried at the temperature of 105±5°C, and then mixed with the bacterial suspension with urea concentration of 0.67 mol/L. The solid-liquid ratio of the mixture was 1:0.3. After that, 120 g of the mixture was filled into a self-designed moulding cylinder, as shown in Fig 1. The moulding cylinder consists of a split PVC tube with inner diameter of 36 mm and height of 80 mm, a layer of geotextile lined between the sample and the PVC tube, and two porous stones placed on the bottom and top of the PVC tube, respectively. The PVC tube was perforated to inlet adequate oxygen for microbial activity. After filling and compacting of the mixture, the moulding cylinder was placed in an incubator with a temperature of 20±2°C and a humidity of ≥95% for curing.

(2) After curing for 24 hours, the moulding cylinder was removed, and the moulded sample column was continued to cure for 6 days. Subsequently, the unconfined compression test was conducted on the solidified sample by using a servo mechanical press (CMT4000, China) with a compressive strain rate of 2 mm/min. The compressive strength was determined by using the formula: P = F/A, where P is the compressive strength (MPa), F is the maximum load recorded at the point of fracture (N), and A is the area of loaded surface (mm^2^).

(3) After the completion of unconfined compression test, the sample was collected and dried at the temperature of 105±5°C for 24 hours, and then pulverized by a rubber mallet. Subsequently, the particle size distribution and the leachable metal content were analyzed using the pulverized sample. The testing methods were in accordance with those described in section “Characteristics of MSWI fly ashes”.

All the experiments were conducted three times to check the repeatability and quantify the changes to the fly ash properties.

### Evaluation of heavy metal leaching toxicity

To evaluate the heavy metals immobilized in the fly ash after solidification and stabilization treatments, a stabilization rate is defined as follows:

$$R=\frac{C_{0}‐C_{f}}{C_{0}}\times 100\%$$ (3)

where, *R* is the stabilization rate of heavy metal; *C*_*0*_ and *C*_*f*_ refer to the leaching concentration of heavy metal before and after the treatment, respectively.

### SEM-EDS analysis

The small-size blocky samples of MSWI fly ash after solidification and stabilization treatments were used to analyze the morphological structures and elemental composition information by using a scanning electron microscope (SEM) equipped with energy dispersive spectroscopic (EDS). The SEM-EDS used was a FEI Quanta 650 FEG ESEM operated in electron detection mode with high-vacuum and an acceleration voltage of 10~20 kV.

## Results and discussion

### Characteristics of MSWI fly ashes

#### Particle size distribution

**Fig 2:**
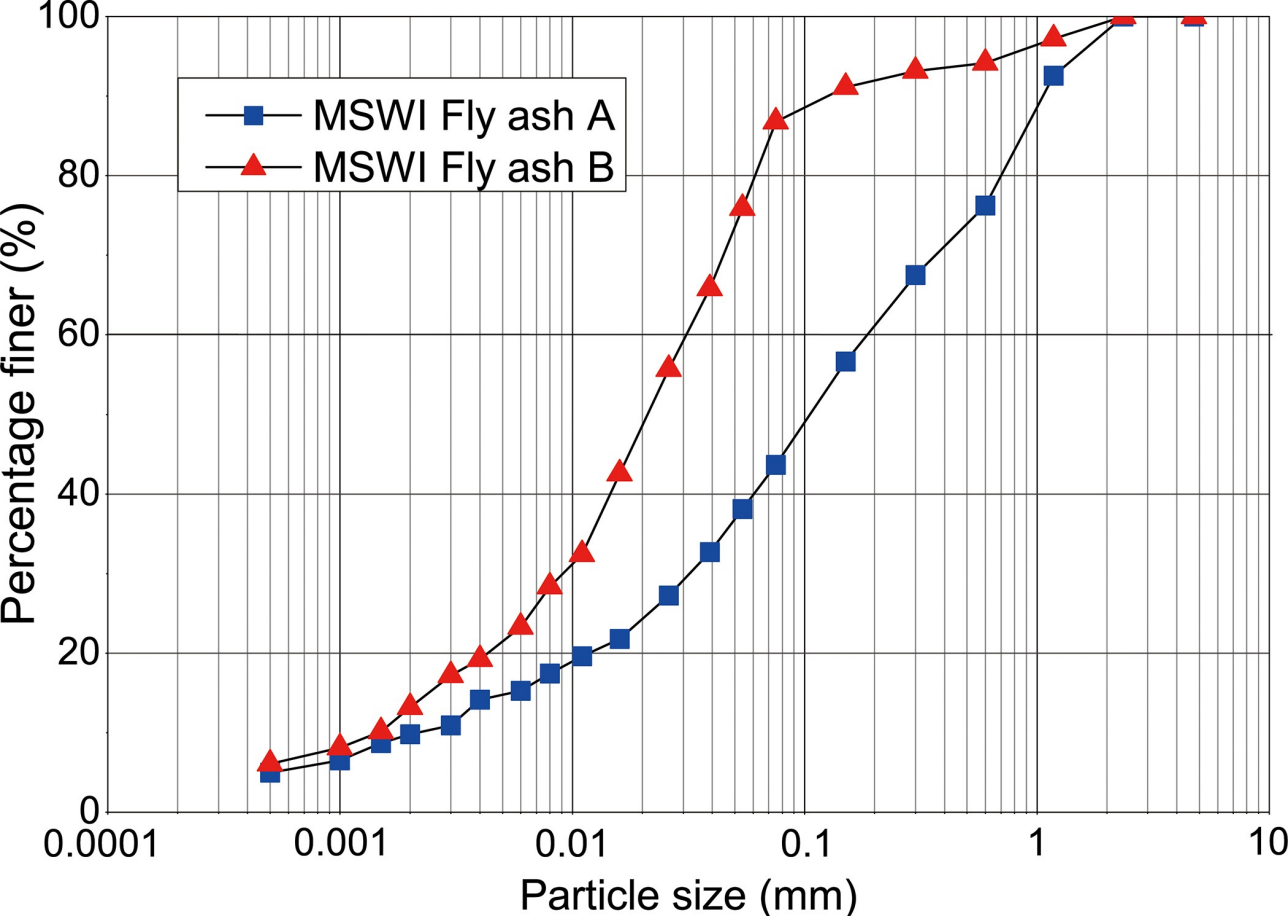
Particle size distributions of fly ashes A and B.

The particle size distributions of MSWI fly ashes A and B are shown in Fig 2. The average particle size of fly ash A was about 0.108 mm and 56.4% of the particles fell within the sand range (0.075 mm < d <2 mm). As for fly ash B, the average particle size was about 0.021 mm and 63.5% of the particles fell within the silt range (0.005 mm< d <0.075 mm). The results showed that fly ash B contained significantly more finer particles than fly ash A. Tang et al [26] reported an average particle size of about 0.11 mm for the fly ash collected from a MSWI facility in Suzhou, China, which was comparable to that of fly ash A in present study. The particle size of the fly ash sampled by González et al [8] from a MSWI plant was very fine with an average value of 0.01~0.02 mm, which was comparable to that of fly ash B in present study. The difference in the particle size of MSWI fly ash might be attributed to the different waste compositions and incineration processes [4].

#### Chemical composition

The chemical compositions of fly ashes A and B are presented in Table 2. The principal constituents were CaO, Cl, SiO_2_, Al_2_O_3_, Fe_2_O_3_, Na_2_O and K_2_O. It was observed that the alkaline oxides (e.g., CaO, Na_2_O, K_2_O, MgO, Fe_2_O_3_) collectively accounted for nearly 60% of the composition. The high content of alkaline oxides resulted in a strong alkalinity of the fly ash. The pH values of the ash-water mixture were obtained as 11.5 and 11.6 for fly ashes A and B, respectively. The contents of CaO were as high as 34.4% and 44.1% for fly ashes A and B, respectively. Hence, there was a sufficient supply of endogenous Ca in the fly ash for MICP treatment. The collectively amounts of SiO_2_, Al_2_O_3_, Fe_2_O_3_ and CaO were 42% and 69% for fly ashes A and B, respectively. Comparable results of the chemical composition of MSWI fly ash have been reported in many papers [26–27]. It was reported in the reference [28] that the fraction of amorphous phases of fly ash was as high as 77.3%. Hence, it is inferred that there is a potential property of self-cementation for MSWI fly ash in the presence of water.

**Table 2:** Chemical compositions of fly ashes A and B (%).

Fly ash	CaO	SiO_2_	Fe_2_O_3_	Al_2_O_3_	MgO	Na_2_O	K_2_O	SO_3_	P_2_O_5_	Cl	Others
**A**	34.39	3.42	1.85	2.58	2.34	11.38	7.71	5.33	3.37	24.31	3.32
**B**	44.07	9.82	5.47	9.85	3.13	3.99	3.19	2.89	3.56	10.32	3.71

#### Leaching concentrations of heavy metals

Table 3 shows the total and leaching concentrations of heavy metals for the fly ashes, together with the leaching limits of the identification standard for hazardous wastes (GB 5085.3–2007). Evidently, Zn was detected to have the highest total concentrations of 9411.2 mg/kg and 4582.0 mg/kg for fly ashes A and B, respectively. Cu and Pb also have high concentrations ranging from 901.2 mg/kg to 1438.9 mg/kg, and followed by Cr, Cd, Ni and Hg. These values are quite comparable with those found in previous studies of MSWI fly ash [3, 8, 23]. It was observed that Zn had the highest leaching concentrations of 4078.4 mg/kg and 3486.5 mg/kg for fly ashes A and B, respectively, and followed by Cu, Pb, Cr, Cd, Ni and Hg. The calculation results showed that the leaching rates of heavy metals were mostly in the range of 10%~76%. It was noted that the leaching concentrations of Zn, Pb, Cr, Hg in fly ash A and Zn, Cu, Pb, Cr, Cd in fly ash B exceed the limits in the standard. This indicated that both fly ash A and B belonged to hazardous waste. Therefore, stabilization of heavy metals in the MSWI fly ash is necessary whether they are going to be reused or properly stored in landfills.

**Table 3:** Total and leaching concentrations of heavy metals (mg/kg).

Items	Zn	Cu	Pb	Cr	Ni	Cd	Hg
A*	9411.2	1438.9	1343.5	296.5	53.6	60.0	3.2
B*	4582.0	1400.1	1310.8	147.7	46.0	127.8	2.6
**A0**	4078.4	824.4	675.9	50.6	5.6	7.6	1.7
**B0**	3486.5	901.2	421.6	100.2	5.0	13.4	0.3
**Limits**	1000	1000	50	50	50	10	1

Notes: A* (B*) and A0 (B0) refer to the total concentration and leaching concentration of heavy metals of fly ash A (B), respectively.

### Microbial concentration assessment

**Fig 3:**
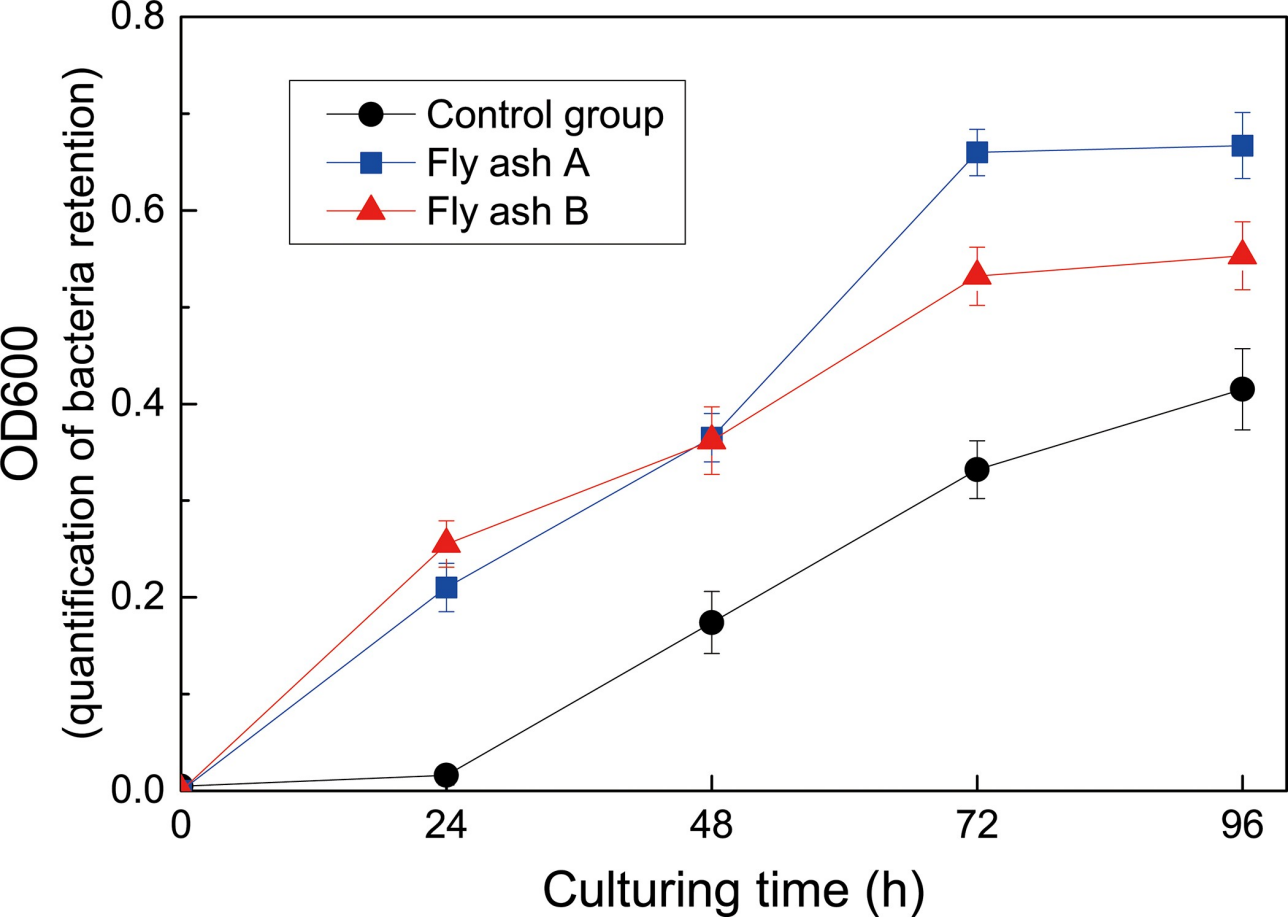
Growth curves of *S*. *pasteurii* incubated under different conditions.

Fig 3 shows the temporal variations of OD600 values of bacterial suspensions cultured in the presence of fly ash A or B, together with the reference group without adding fly ash. It was observed that the OD600 values slightly increased to 0.02 in the first 24 hours, and then rapidly increased to 0.42 in the following 72 hours for the reference group. The OD600 values of the cases containing fly ash A and B rapidly increased to 0.66 and 0.53 in the first 72 hours, respectively, and further increased to 0.67 and 0.55 in the following 24 hours. The results showed that *S*.*pasteurii* grew even better in the presence of MSWI fly ash. This interesting result indicated that the high levels of heavy metal and alkalinity did not inhibit the activity of *S*.*pasteurii*, but promoted its growth to some extent. A number of papers have reported that the microbes could survive highly alkaline and heavy metal toxic environment caused by the presence of fly ash, and sometimes its activity was even higher at that environment [29–31]. This might be attributed to the fact that the fly ash at moderate levels provides nutrients or elements that are favorable to the microbial activities [32–33]. Moreover, Bachmeier et al [34] has found that the addition of nickel (5~100 μmol/L) would promote the bacteria activity. In this study, the nickel concentration was obtained as 8.5~9.5 μmol/L, which tended to be beneficial to the activity of *S*.*pasteurii*.

In present MICP experiments, the bacterial suspension cultured for 48 hours was adopted for the solidification and stabilization treatments of fly ash samples. The OD600 value of the bacterial suspension was 0.174±0.032, which was estimated to a bacterial concentration of 7.93±0.79×10^6^ cells/mL. It should be noted that the bacterial concentration is slightly lower than those reported in literatures [24, 35]. The lower bacterial concentration in the suspension is probably attributed to the dilution effect of supernatant liquid which was used to measure the OD600 value. Martin et al [36] has observed a significant activity of *S*.*pasteurii* at the low OD600 of about 0.14. Jiang et al [18] has also reported that OD600 value of the final suspension ready for MICP treatment is 0.454 ±0.137. From the above views, it is believed that the bacterial concentration in present work was sufficient to induce ureolytic reactions.

### UCS of MICP treated fly ashes

**Fig 4:**
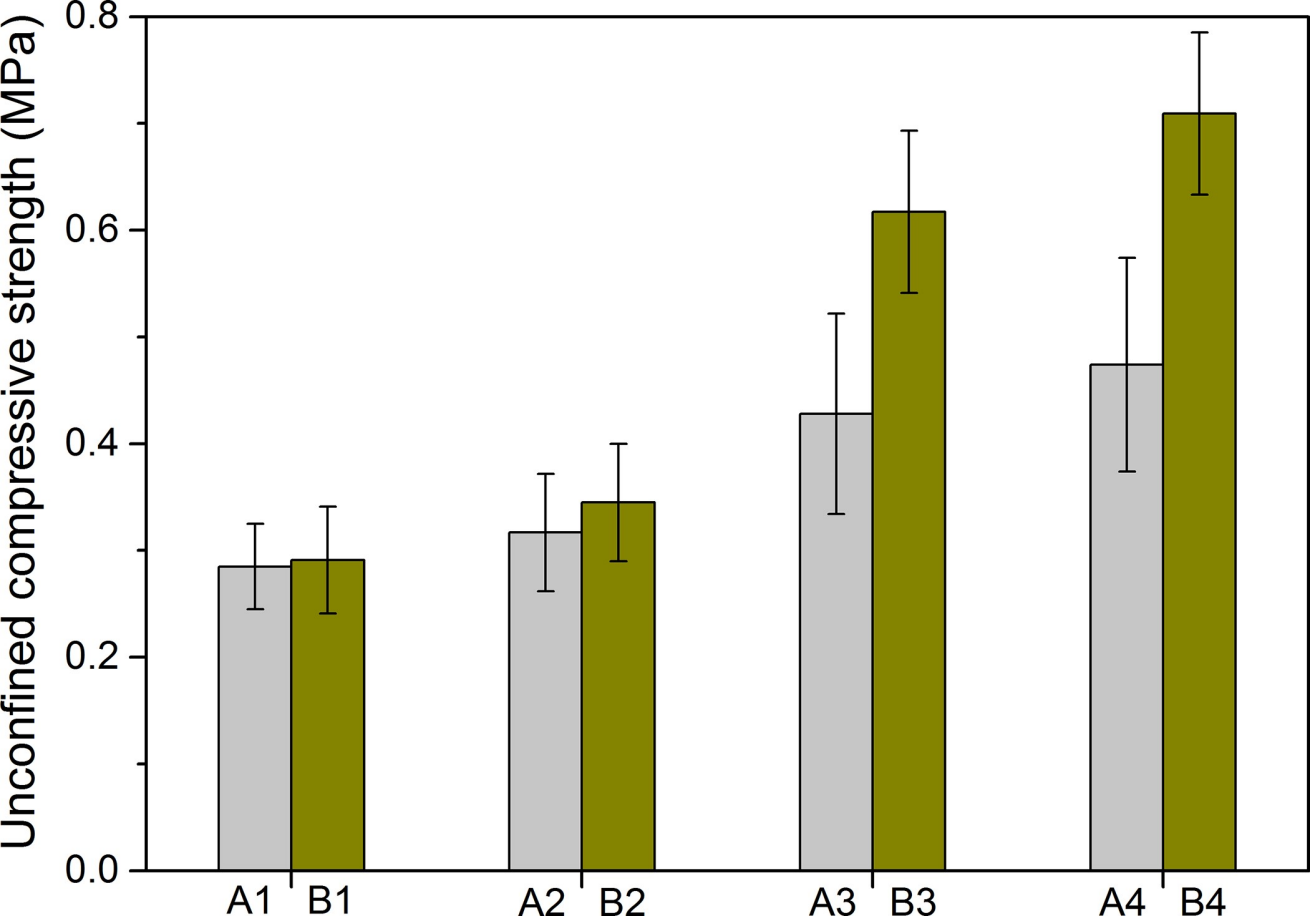
UCS of the treated MSWI fly ashes.

Fig 4 shows the UCS values of the fly ashes A and B after different treatments. When mixing the fly ash B solely with deionized water, being the reference group B1, the compressive strength after self-cementation was obtained as 0.284 MPa, which is quite comparable to that reported in the references [37–38]. This was mainly attributed to the calcium silicate hydrate and calcium aluminate hydrate produced from the hydration reactions of calcium silicate and calcium aluminate embodied in the fly ash. When mixing the fly ash B with urea solution, being the case B2, the compressive strength increased to 0.343 MPa, which was 21% higher than the reference group B1. This occurrence might be associated with the slow self-hydrolysis of urea for B2 [39], which would result in a small amount of calcium carbonate crystal, as described in Eqs (1) and (2). When mixing the fly ash B with bacterial suspension (B3), the compressive strength further increased to 0.617 MPa, being 117% higher than that of B1. The maximum compressive strength was belonged to B4, which had a higher urea concentration in the bacterial suspension than B3. The compressive strength of B4 was obtained as 0.709 MPa, which was about 150% greater than the reference group B1. The above results indicated that the fly ash B after MICP treatments (B3 and B4) has a significant improvement in compressive strength with respect to the untreated one (B1). This can be explained by the fact that an introduction of urease-producing bacteria was beneficial to accelerate the processes of urea hydrolysis and subsequent calcium carbonate precipitation.

Similar testing results of B1-B4 were also observed in the cases A1~A4, as shown in Fig 4. However, the compressive strength obtained from fly ash B was greater than that from fly ash A under the same treatment condition. For example, the compressive strength of A4 was obtained as 0.385 MPa, being 47% lower than that of B4. Two primary reasons could be involved to explain this. Firstly, the collectively amount of SiO_2_, Al_2_O_3_, Fe_2_O_3_ and CaO was greater in fly ash B than that in fly ash A (Table 2), which resulted in much more binding materials for solidification for the former. Secondly, the grain size of fly ash B was significantly finer than that of fly ash A, which was probably associated with the fact that much more not completely burnt residues were contained in fly ash A [23]. However, the residues can influence the compressive strength of solidified fly ash by reducing the binding material for cementation and also destroy the structure of hydration products [23]. The reason for this is that the residues are porous and hydrophobic. Thus, the binding material can easily be wrapped in the inner of the carbon hole of the residues to form a protective film, which impedes the full contact of water with binding material [26].

Li et al [40] found that the 8-day UCS value was 1.21 MPa for MSWI fly ash with cement content of 10%, and increased to 2.49 MPa with 20% cement. Polettini et al [41] presented the results that the 7-day UCS value reached over 6 MPa for the MSWI fly ash with cement dosage up to 20%. It is observed that MSWI fly ash solidified by cement could attain higher UCS values than that treated by MICP, which is mainly attributed to the high bonding strength of cement. Huang et al [38] reported that the 28-day UCS value of the mixture at a ratio of MSWI fly ash to phosphor powder of 90:10 was 0.4 MPa, being 33% higher than that without adding phosphor powder. This UCS value is slightly lower than that solidified by MICP in this study, suggesting weaker cementing capacity for phosphor powder.

### Particle size distribution of MICP treated fly ashes

**Fig 5:**
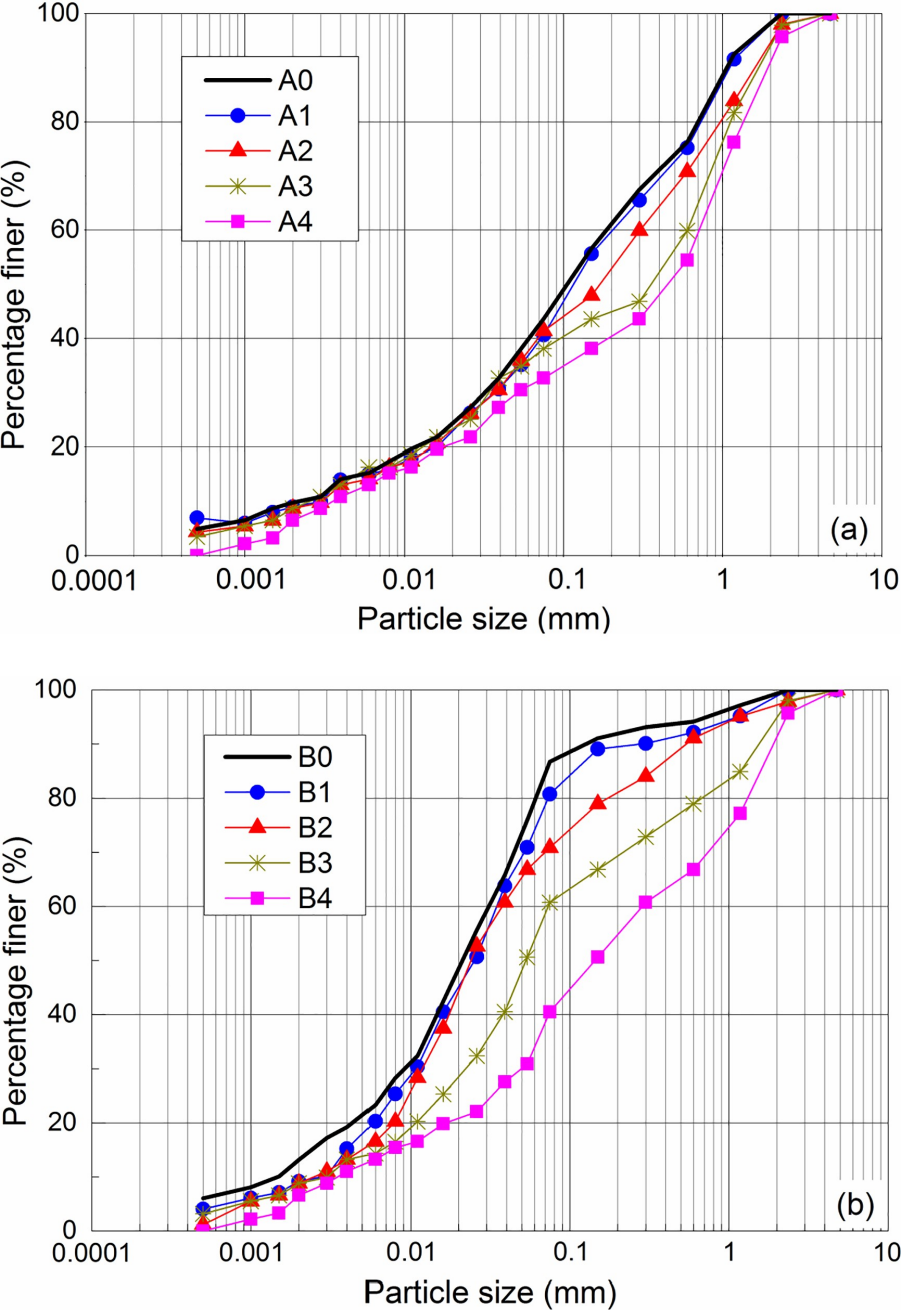
Particle size distributions of (a) fly ash A and (b) fly ash B before and after treatment.

Fig 5(A) and 5(B) shows the measured particle size distribution curves of the fly ashes after different treatments. The cases A0 and B0 refer to the original fly ashes A and B, respectively. It was observed that the particle size of the treated fly ashes tended to be greater than that of untreated ones. When comparing with B0, B4 increased most in the particle size, followed by B3, B2 and B1.This sequence was consistent with the improvement in compressive strength. Similar results were also observed in fly ash A. In general, the fly ash B exhibited a greater increase in particle size than fly ash A for the cases under the same treatment condition.

**Fig 6:**
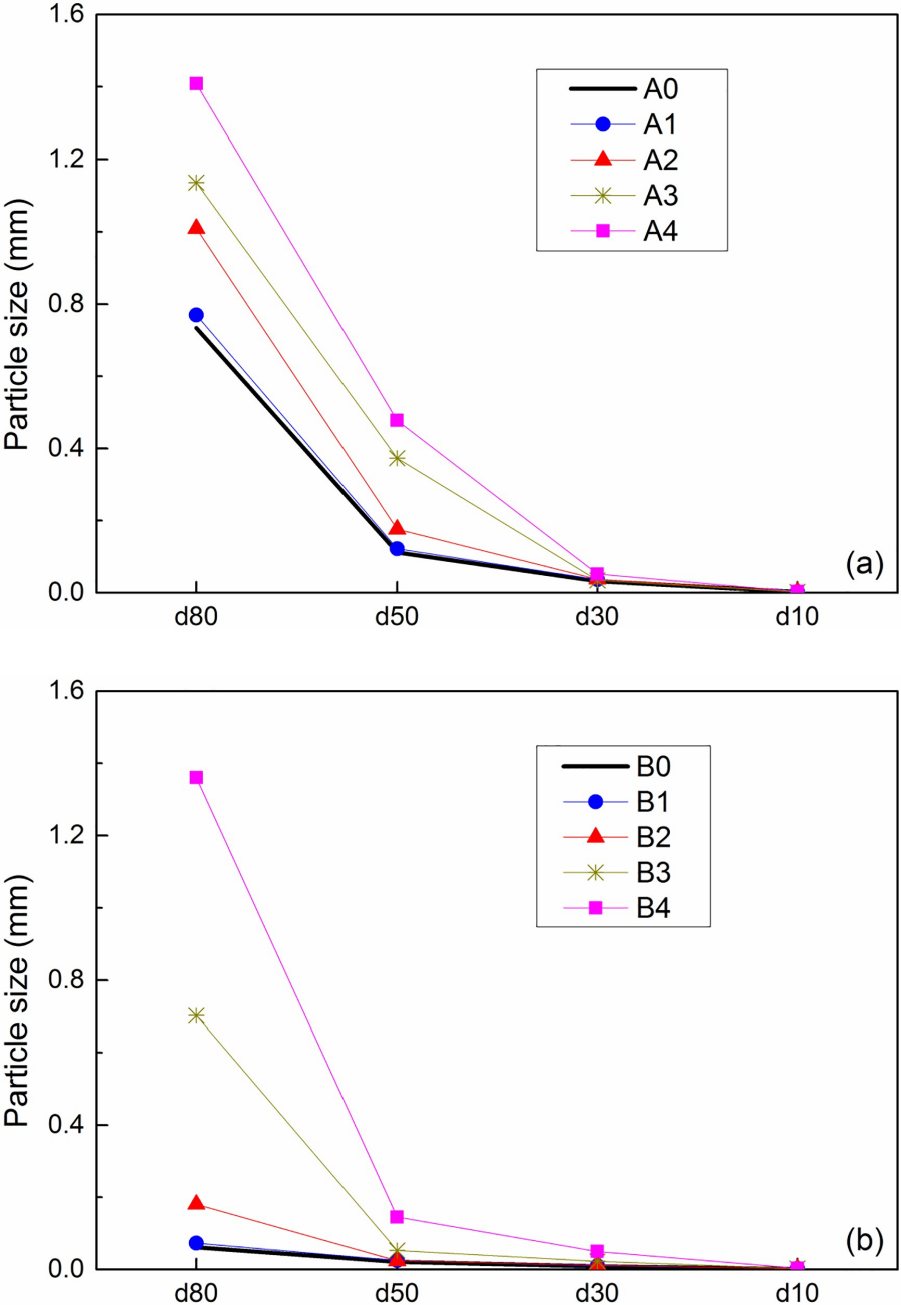
Changes in particle sizes of the treated (a) fly ash A and (b) fly ash B.

To present a more intuitive analysis of the change in particle size of the fly ashes before and after the treatments, the parameters *d*_80_, *d*_50_, *d*_30_ and *d*_10_ were calculated by using the particle distribution curves in Fig 5(A) and 5(B). Here, *d*_80_, *d*_50_, *d*_30_ and *d*_10_ refer to the diameter values corresponding to 80%, 50%, 30%, and 10% of the particle sizes smaller than this value, respectively. The calculated particle size parameters for fly ashes A and B are shown in Fig 6(A) and 6(B). It was observed that the fly ash A followed the order of A4 > A3 > A2 > A1 > A0 in the particle size at a given parameter of d_80_, d_50_, d_30_ or d_10_. Here taking the mean diameter d_50_ for example. The value of d_50_ was obtained as 0.112 mm for the original fly ash A0. As for the treated fly ashes A1, A2, A3 and A4, the values of d_50_ were 0.122, 0.176, 0.373 and 0.477 mm, respectively, being 9%, 57%, 233% and 326% greater than that of A0. Similar trends were also observed in fly ash B. The values of d_50_ for B1, B2, B3 and B4 were 12%, 17%, 145% and 571% greater than that of B0.

The above results indicated that the treatments of MSWI fly ash resulted in an increase in the particle size. This effect was most significant in the cases 3 and 4 after MICP treatment, and followed by case 2 and case 1. This sequence was consistent with the improvement in the UCS. During the solidification process of the fly ash, the produced calcium silicate hydrate and calcium-carbonate tended to wrap around the fine particles and bind the other particles together to form larger particles. These two effects, binding and wrapping, might be the reasons of particle size increasing. The effect of particle size increasing was also observed in the MSWI fly ash treated by other materials. Xu et al [42] reported that the d_50_ of MSWI fly ash stabilized and solidified by using an organic chelator and lime was 7~20 times of the original fly ash, this value is much larger than that treated by MICP in this paper. This is thought to be related to the much significant effects of binding and wrapping in the former one.

### Stabilization of heavy metals

**Fig 7:**
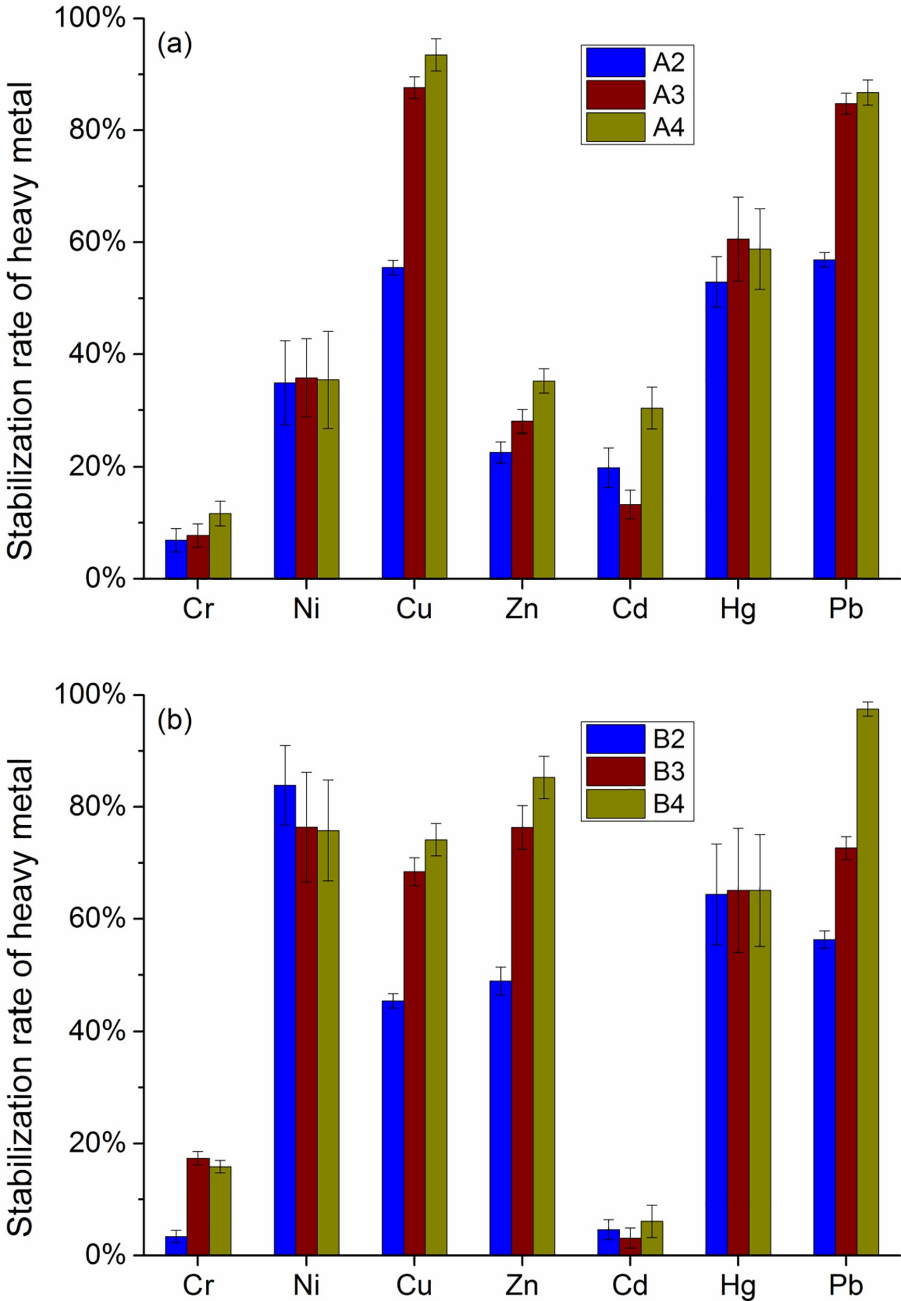
Stabilization rate of heavy metals for the treated (a) fly ash A and (b) fly ash B.

Fig 7(A) and 7(B) shows the stabilization rates of heavy metals in MSWI fly ash after different treatments. The stabilization rate was adopted to evaluate the amount of the heavy metal stabilized in the treated specimens. As for fly ash A, the stabilization rates of Cu, Hg and Pb were 55.5%~93.5%, 52.9%~60.6% and 56.9%~86.7%, respectively. However, the stabilization rates of Zn, Ni, Cr and Cd were relatively lower, being 22.6%~35.2%, 34.9%~35.8%, 6.9%~11.7% and 13.3%~30.4%, respectively. The results indicated that Cu, Hg, Zn, Ni and Pb were better stabilized than Cr and Cd in the fly ash A. As for fly ash B, the stabilization rates of Ni, Cu, Zn, Hg and Pb were 75.8%~83.9%, 45.3%~74.1%, 48.9%~85.3%, 64.4%~65.1% and 56.3%-97.5%, being much higher than that of Cd (3.1%~6.1%) and Cr (3.4%~15.9%). It was observed from the two types of fly ashes that the case 4 gained a largest stabilization rate for most of the heavy metals, followed by the case 3 and case 2. This indicated that the MICP treatment was able to well stabilize the heavy metals contained in the MSWI fly ash. It was also observed that the fly ash B, which has finer particle sizes and a higher Ca content than fly ash A, had a higher stabilization rate for most of the heavy metals after MICP treatment.

Tang et al [26] investigated the stabilization behavior of MSWI fly ash by using the cement as a binder at cement/fly ash ratio of 4:6, and found that the stabilization rates of Pb, Cu, Cd, Cr and Ni were 95.9%, 48.4%, 98.6%, 29.3% and 78.8%, respectively. Bie et al [43] reported that the stabilization rates of Pb, Cu, Cd, Cr and Zn were 27.0%, 20.0%, 75.0%, 84.4% and 30.0%, respectively, for the mortar specimens blended with 80% MSWI fly ash and 20% cement at pH = 8.0. Zhao et al [3] found that the stabilization rates of Pb and Cr were over 80% and 92%, respectively, for MSWI fly ash solidified by 33% (of fly ash by weight) cement or 33% asphalt, and were higher than 89% and 92% respectively when stabilized by 5% sodium sulfide or 4% thiourea. Jiang et al [44] used a chemical chelating agent to stabilize the heavy metals in MSWI fly ash, and observed that the stabilization rates of Pb, Cd, Zn and Cr reached over 85% at a chemical dosage of 0.2% by weight, and can be higher than 90% at chemical dosages of 0.4% and 0.6%. Gong et al [45] used thermal treatment to stabilize MSWI fly ash, and found the stabilization rates of Pb, Cu and Zn were 86.1%, 66.7% and 56.4%, respectively, at 700 ^o^C treating for 10 min. It is observed that MSWI fly ash treated by MICP could achieve comparable stabilization rates for Pb, Cu and Zn, and slightly lower stabilization rates for Cd and Cr, when compared with the above solidification/stabilization methods. It is reported that Cr and Cd cannot be effectively stabilized when pH is over 10.5 [46–47], and given the high-alkaline environment during MICP process in this study, this can help explain why the stabilization rates of Cr and Cd are not very efficient.

### SEM-EDS analysis

**Fig 8:**
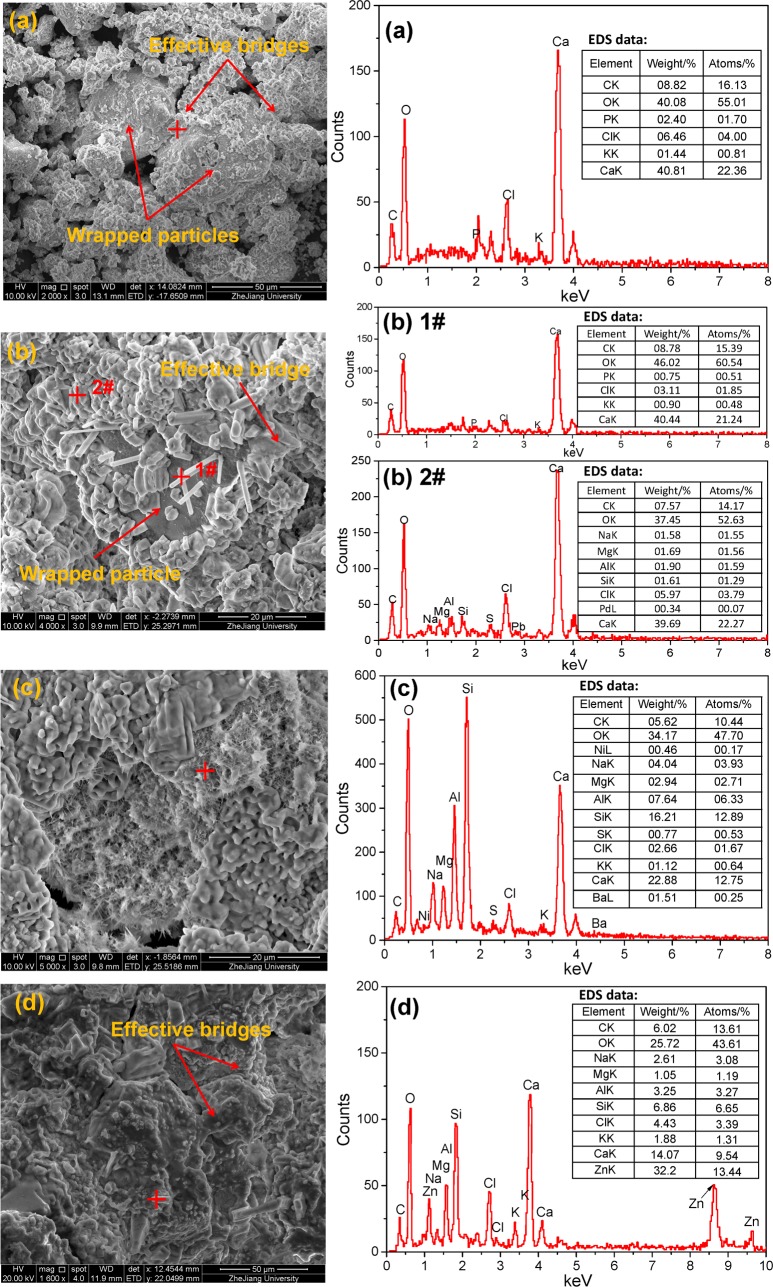
SEM micrographs and EDS spectrums (marked + in left figure) of MICP treated MSWI fly ashes.

Fig 8(A)–8(D) shows the SEM micrographs and EDS spectrums of stabilized MSWI fly ash (B4), the EDS spectrums were determined at the position marked “+” in the SEM micrographs. It was seen from the SEM micrographs in Fig 8(A)–8(D) that a significant amount of crystals was formed and wrapped at the surface of fly ash particles, while the crystals formed bridges to link the neighboring particles together. This would result in an increase in particle size and an enhancement in compressive strength for solidified fly ash. The formed crystals have a various shapes and are determined as different minerals. The acicular crystals observed in Fig 8(B) (marked “+1#”) and the hexahedral crystals in Fig 8(B) (marked “+2#”) had similar elementary compositions, which were mainly composed of Ca, O, C, etc. These acicular and hexahedral crystals were probably aragonite and calcite, respectively, both of which were similar to those observed and confirmed in Zhang et al [48]. The fibriform and reticular gels/crystals observed in Fig 8(C) (marked “+”) were mainly composed of Ca, O, C, Al, Si, etc., indicating the possible presence of calcium silicate hydrate and calcium aluminate hydrate [49]. The trace heavy metals detected in the above EDS spectrums, e.g., Pb and Ba, might be associated with the physical encapsulation of the binding materials (i.e., calcium carbonate, calcium silicate hydrate and calcium aluminate hydrate) and the physical absorption of the fly ash particles. The subulate/cylindrical crystals observed in Fig 8(D) (marked “+”) were mainly composed of Zn, O, C, Ca, Si, Al, etc. From the perspective of atomic percent, Zn: C: O = 13.44:13.61:43.61, which was close to the chemical formula of zinc carbonate, hence there was a certain possibility for the presence of zinc carbonate. Further, the shape of the crystal was quite similar to the zinc carbonate that observed and confirmed in Li et al [50]. This result indicated that there is a high possibility that the heavy metals contained in fly ashes might be transformed into carbonate after MICP treatment, which has stronger stability and lower toxicity compared with heavy metal ions. The above observations can give evidences for the explanations of the enhancement in compressive strength, the increase in particle size and the stabilization of heavy metals of the MICP treated MSWI fly ashes.

## Conclusions

Both MSWI fly ashes A and B contained multiple types of heavy metals. The leaching concentrations of Zn, Pb, Cr, Hg in fly ash A and Zn, Cu, Pb, Cr, Cd in fly ash B exceed the limits of the identification standard for hazardous wastes. It is necessary to stabilize the heavy metals in the MSWI fly ash. Positively, the high CaO contents (i.e., 34.4% and 44.1%, respectively) of both fly ashes A and B can create a sufficient supply of endogenous Ca for the MICP treatment, which is vital during this process.

At an ash-water ratio of 1g:10 mL, the leaching solution from each fly ashes was strongly alkaline (pH = 11.5~11.6) with high toxicity of heavy metals (Cu, Zn, Cd and Pb). It is proven that bacteria *S*. *pasteurii* can survive from fly ash solution of high alkalinity and heavy metal toxicity with the capability to tolerate severe environment.

The MICP treatment resulted in a significant increase in the UCS and particle size. After 7 days MICP treatment, the UCS value of fly ashes A and B reached 0.385 MPa and 0.709 MPa, and the average particle size of fly ashes A and B extended from initial 0.108 mm and 0.021 mm to 0.477 mm and 0.145 mm, respectively.

The MICP treatment attained a noticeable reduction in the leaching toxicity of heavy metals, especially for Cu, Pb and Hg. Fly ash B, which had finer particle sizes and a higher Ca content than fly ash A, ended up with a higher stabilization rate of heavy metals.

These findings above shone a light on the possibility of using MICP technique as a suitable and efficient tool to treat the MSWI fly ash before being reused or properly stored in landfills. However, to enable the MICP technique to be applied in practical implementations with greater confidence, more work should be done in the future, e.g., the long-term leaching behavior of heavy metal in MICP treated fly ash, the dynamics of the calcium dissolution process.

## Supporting information

S1 Data Data set for particle size distributions of fly ashes A and B. (OPJ)

S2 Data Data set for growth curves of *S*. *pasteurii* incubated under different conditions. (OPJ)

S3 Data Data set for unconfined compressive strengths of the treated fly ashes. (OPJ)

S4 Data Data set for particle size distributions of fly ash A before and after treatment. (OPJ)

S5 Data Data set for particle size distributions of fly ash B before and after treatment. (OPJ)

S6 Data Data set for changes in particle size of fly ash A after treatment. (OPJ)

S7 Data Data set for changes in particle size of fly ash B after treatment. (OPJ)

S8 Data Data set for stabilization rate of heavy metals for the treated fly ash A. (OPJ)

S9 Data Data set for stabilization rate of heavy metals for the treated fly ash B. (OPJ)
